# Words matter: measuring and titrating the communicative character of AI

**DOI:** 10.3389/frai.2026.1815425

**Published:** 2026-09-03

**Authors:** William C. Kouns

**Affiliations:** Independent Researcher, Knoxville, TN, United States

**Keywords:** communicative character, large language models, lexical analysis, human-AI interaction, prompt engineering, AI governance

## Abstract

As large language models become primary communication partners, the stylistic and communicative character of their output—specifically the degree to which it relies on intellectual, affective, or action-oriented language—shapes how users interpret, and act on what they read. Yet this property is rarely measured or controlled directly. We introduce the Intellect-Emotion-Action Profile (IEAP), a purpose-built lexical framework featuring an inductively constructed dictionary from AI-generated text. IEAP decomposes any response into the proportional usage of intellectual, affective, and action words. Using a single automated harvester, we elicited and scored responses from four contemporary architectures (Claude, ChatGPT, Grok, and Gemini) across five question domains under four instructional-mode directives that span a cold-to-fire register (*n* = 30 per architecture per domain). Three primary findings emerge. First, when no directive is applied, native communicative profiles are dominated by the underlying question, with architecture playing a secondary, architecture-dependent role; thus, the prompt establishes the baseline for the response. Second, an explicit mode directive displaces this profile substantially from its baseline. Specifically, the directives produced large, monotonic shifts in affective word usage, exceeding 50 percentage points in the most responsive domain with no reversals. Third, response depth alters length and conceptual breadth while leaving lexical composition essentially unchanged, establishing register and depth as orthogonal controls. These three dimensions correlated with human-rated NRC lexicons in the expected directions, supporting their validity. Within-domain robustness checks reproduce both the baseline profiles and the titration trajectories across multiple question framings. Because a measurable communicative property can be monitored and audited, these results position IEAP as a practical foundation for studying, comparing, and ultimately governing how AI systems communicate.

## 1 Introduction

When humans communicate, we do not simply transmit information. We blend analytical reasoning, emotional connection, and calls to action in proportions that vary with context, audience, and purpose. A physician explaining a diagnosis leads with intellectual precision; a friend offering comfort leads with emotional presence; a coach driving performance leads with action directives. The mix matters because it shapes how the listener processes, trusts, and responds to the message. As large language models (LLMs) become primary communication partners in healthcare, education, and professional settings, the ability to measure and govern this communicative mix is essential.

Whatever computations occur inside a language model, humans encounter the system only through its words. Words constitute the entire interface between an AI system and its user—the channel through which reasoning, emotion, and direction are conveyed, interpreted, trusted, and acted upon. The communicative character of an AI system is therefore not hidden in its architecture but is instead expressed and made consequential in its output text. This is the premise of the present study: because communicative characters are formulated and delivered through language, language is precisely where they can be observed, compared, and deliberately shifted (Rahwan et al., 2019).

This study examines with LLM-powered systems that interact with users through natural language, such as Claude, ChatGPT, Grok, and Gemini. While the underlying framework may extend to other modalities, the present study is limited to text-based interaction; multimodal, embodied, and voice-based systems are beyond its scope.

Existing approaches to analyzing textual and conversational content fall into several traditions, none of which directly address the measurement and instructional control of communicative character in AI output. Research on conversational interaction has established that human language production is fundamentally adaptive: speakers continuously coordinate with their interlocutors and adjust their language to the listener and the social context—processes known as audience or recipient design and supported by social-cognitive mechanisms (Clark, 1996; Brennan and Clark, 1996; Horton and Keysar, 1996; Kuhlen and Abdel Rahman, 2023). Communication theory has long decomposed this adaptivity into functional components: Jakobson's (1960) taxonomy of language functions distinguishes referential, emotive, and conative orientations, while signaling accounts separate the information-transfer and social-influence components of communicative acts (Mehu, 2015). Although these frameworks describe how and why communicative character varies, they remain theoretical accounts of human communication that do not provide an instrument for measuring that character in AI-generated text.

The analysis of textual content has produced mature lexical instruments for quantifying the psychological and communicative dimensions of language. The most widely used instrument, Linguistic Inquiry and Word Count (LIWC), assigns words to predetermined, psychometrically validated categories spanning cognitive, affective, and social processes, reporting their relative frequencies in a text (Tausczik and Pennebaker, 2010). Sentiment and emotion lexicons, including VADER (Hutto and Gilbert, 2014) and the NRC lexicons (Mohammad and Turney, 2013; Mohammad, 2018a, 2025a), provide complementary measures of valence, arousal, dominance, and emotion. Furthermore, cognitive-affective-behavioral formulations of engagement have been applied to human-authored texts in educational and reader-response research (Fredricks et al., 2004; Ellis, 2010). These instruments are powerful, but their categories are derived from human language corpora and psychological constructs, measuring sentiment or trait content rather than the compositional communicative register of a text (Biber, 1988). Crucially, none were built from or calibrated to AI-generated language.

A third tradition, prompt engineering for LLM-based systems, has demonstrated that the framing of an instruction substantially shapes model output. Chain-of-thought and related strategies improve reasoning (Wei et al., 2022; Kojima et al., 2022), while broader model output is highly sensitive to prompt format, ordering, and framing, exhibiting measured swings in behavior based on subtle wording variations alone (Reynolds and McDonell, 2021; Lu et al., 2022; Zhao et al., 2021). Recent studies even treat prompt framing explicitly as a tunable interface parameter (Tuladhar and Kinney-Lang, 2026; Yang and Caput, 2025). A closely related body of study induces personality in LLMs through prompting, typically using human personality frameworks such as the Big Five to steer models toward trait poles (Serapio-García et al., 2025; Jiang et al., 2024). While this literature establishes that instruction controls output, it has largely focused on task performance or personality trait induction rather than on the communicative composition of the response. Crucially, it has not provided a framework to measure that composition against which framing effects can be quantified.

Finally, a growing body of literature addresses the implications of registration in human–AI interaction. Applied lexical tools have been used to characterize AI communication in healthcare contexts (Biassoni and Gnerre, 2025), and reception studies show that communicative style, and not only content, shape outcomes. Furthermore, AI-augmented messaging has been shown to enhance empathic quality in peer support settings (Sharma et al., 2023). Complementary interpretability research characterizes model emotion and persona through internal activation directions, showing that these can be steered or drifted, particularly when interacting with emotionally vulnerable users (Lu et al., 2026)—effectively measuring character from within the model. In contrast, IEAP offers an external, output-level counterpart applicable to black-box commercial systems, raising the open, testable question of whether character expressed in AI-derived text corresponds to the internal directions that interpretability identifies. While prior research confirms that register matters and is, in principle, controllable, it largely treats AI communicative style as a fixed property to be characterized rather than as a quantity to be measured and deliberately titrated.

Across these traditions, a consistent gap emerges. Communication theory describes communicative character but does not measure it in AI; lexical instruments measure human-derived sentiment and traits but are not built for the AI register; prompt engineering and personality-induction techniques control output but do not measure communicative composition; and register studies show that style matters but treat it as a fixed property. The IEAP (intellect-emotion-action) framework fills this gap. Its dictionary of 1,897 terms was constructed inductively from AI-generated responses, creating a purpose-built lexical instrument calibrated to actual AI vocabulary rather than human language norms. Consequently, IEAP decomposes its output into intellectual, affective, and action-oriented components. While psychometric approaches measure borrowed personality traits and interpretability methods read internal activations, IEAP measures the words themselves—the primary site where communicative character is expressed in language.

The theoretical basis for these three dimensions derives from a longstanding tripartite tradition in psychology that distinguishes cognitive, affective, and conative (action-oriented) dimensions of human functioning (Hilgard, 1980)—a structure recurring throughout attitude theory and communication research (Breckler, 1984; Eagly and Chaiken, 1993). The IEAP framework does not propose a new theory of mind. Rather, it adapts this established tradition to written communication by operationalizing intellectual, affective, and action-oriented language as observable communicative functions. Crucially, it makes no claims about the internal states of the systems it measures, a question that remains the subject of active debate (Shanahan, 2024; Bender and Koller, 2020; Attah, 2025; Shanahan et al., 2024). Its implementation is explicitly linguistic, measuring surface patterns in language rather than latent psychological states.

Contemporary studies continue to treat communicative content as decomposable along these same lines. Recent text-analytic research operationalizes cognitive, affective, and behavioral engagement as distinct, separately coded dimensions of written communication (Zimmerman et al., 2025), mirroring IEAP’s intellectual, affective, and action structures, whereas neurocognitive evidence indicates that words engage distinct neural systems, with action-related language recruiting sensorimotor regions rather than language areas alone (De Stefani and De Marco, 2019). IEAP operationalizes this convergent structure at the level of word usage.

Future adaptive human-AI systems may incorporate supervisory layers that monitor conversational state and determine, turn by turn, whether a response should remain analytical, become more emotionally supportive, or offer greater procedural guidance—adapting to the user’s momentary needs rather than holding a single configuration fixed for an entire conversation. Such systems first require an operational method for measuring communicative character and evidence that it can be deliberately shifted. Measurement is also a precondition for governance. As the same capability that tunes communicative character for the user’s benefit could also be misused to manipulate users (Carroll et al., 2023), any dimension that can be quantified can also be monitored, audited, and held to account. We do not build such a complete adaptive system here; instead, we establish its necessary precondition: an empirical demonstration, using the IEAP framework, that the intellectual-affective-action composition of AI output can be systematically titrated through instruction. Specifically, we address three questions:

*RQ1*: What is the native communicative profile of AI architectures across question domains?

*RQ2*: Can mode directives reliably titrate communicative characters, and what are the limits of that titration?

*RQ3*: Does titration change the type of language within a dimension, or only the amount?

## 2 Materials and methods

### 2.1 The baseline harvester

All data were collected and scored using a single purpose-built instrument: the SYN-IQ Native Baseline Harvester. The Harvester is an integrated tool that executes the entire pipeline—from controlled elicitation through multi-instrument scoring to structured export—in a single automated run, ensuring that every response in the corpus is produced and measured under identical, version-controlled conditions. Three Harvester revisions were used during data collection (V48, V48bb, and V50); they differed only in elicitation-side infrastructure (provider call configuration, retry handling, and the addition of custom-question slots) and shared a byte-identical scoring path, as verified by exact comparisons of the IEAP dictionary contents and scoring routines across versions.

### 2.2 Controlled elicitation and model instantiation

Harvester constructs each prompt programmatically by composing three layers into a single instruction string in a fixed order: a mode-directive header (the cold, hot, or fire instruction, or none for native), a fixed-depth instruction, and the question text. This order was held constant across all Harvester revisions and was verified by exact comparisons of the prompt-construction routine across versions; prompt order was thus treated as a controlled constant rather than a discretionary formatting choice. This string was submitted as a single user-role turn through each architecture’s native production API; no system role prompt or conversational history was supplied, ensuring that the model was instantiated solely from the composed prompt. Each question was tracked by a content hash to ensure that every architecture received a byte-identical prompt. Consequently, the only manipulated variable was the mode directive, as depth, output-token limits, and all decoding parameters were held constant across conditions. Notably, the named modes (cold/hot/fire/native) were prompt-level register directives, rather than adjustments to the API sampling temperature, which was maintained at each provider’s default and kept identical across all conditions. Each API call was wrapped in retry logic with exponential backoff to absorb transient API failures without contaminating the dataset.

Exact pin model identifiers were recorded for every API call. Claude, ChatGPT, and Grok were held constant across the entire corpus (claude-sonnet-4-20250514, gpt-4o, and grok-3-latest, respectively). For Gemini, the principal question set was collected using gemini-2.0-flash, whereas the custom-question runs were collected using gemini-2.5-flash with model-internal “thinking” disabled (thinking budget set to zero) to ensure that the returned text reflected the model’s direct communicative output, comparable to the non-reasoning output of the other architectures. The Harvester version was recorded alongside each data entry, making the specific Gemini identifier recoverable per response.

Because decoding was stochastic and no fixed random seed was available through these production APIs, each of the *n* runs within a condition (*n* = 30) constituted an independent draw from the same fixed network under a byte-identical prompt. Model parameters and eliciting prompts were held constant; thus, stochastic decoding yielded a distinct yet closely related response to each draw analogous to near-siblings rather than exact copies. This approach was chosen by design: replication within a condition estimates the central tendency and dispersion of a stable response distribution rather than recovering a single deterministic output (Huynh and McNamara, 2026).

### 2.3 Scoring and export

Each returned response was scored in a single pass by several independent instruments. The IEAP dictionary (V3, 1,897 terms) was applied by tokenizing the response and matching tokens against the INT, AFF, and ACT categories to derive a compositional profile. In addition, the Harvester resolved each dimension into its subclasses using the IEAP Subclass Taxonomy (23 subclasses across the three dimensions, loaded from versioned taxonomy files) to yield a within-dimension breakdown. Although computed in-pass for diagnostic purposes, this sub-classification was excluded from the primary exported record for the present study. In the same pass, the Harvester calculated auxiliary metrics: VADER compound sentiment, Flesch–Kincaid grade level, Flesch reading ease, type–token ratio, unique-word count, total word count, and sentence embeddings. Computing all the instruments in a single pass over each response ensured that every metric was derived from identical text.

Every response was stored as a single record carrying its full provenance and derived measures: architecture, mode, depth, question identifier, run index, the three IEAP percentages, auxiliary instrument scores, raw response text, and its embedding. The complete dataset was exported as a structured CSV file that served as the single source of truth for all downstream analyses; every figure and statistic reported herein was traced to a specific row in that export. The instrument’s reproducibility is therefore two-tiered. First, the elicitation protocol is reproducible in a distributional sense: rerunning the pipeline with the same pinned identifiers and fixed parameters regenerates a corpus that is statistically equivalent; individual responses vary but cluster tightly by experimental condition rather than varying randomly. Second, the measurement is reproducible in an exact sense: rescoring any fixed text through the same dictionary version regenerates its IEAP profile deterministically, bit for bit.

### 2.4 The IEAP framework

The Intellect-Emotion-Action Profile (IEAP) scores an AI response by matching its words against three communicative dimensions: Intellectual (INT: analysis, reasoning, and abstraction), Affective (AFF: empathy, emotion, and relational connection), and Action (ACT: procedures, directives, and practical guidance). Words that match none of the three dimensions are excluded from analysis. Each response is tokenized into lowercase word tokens, and INT%, AFF%, and ACT% are each computed as the category word count divided by the total number of matched tokens, multiplied by 100. The three percentages sum to 100% for each response. IEAP creates a compositional measure in which gains in one dimension necessarily offset the others, representing each response as a point on a three-component simplex. Crucially, IEAP is not a sentiment model; it measures how language is distributed across three communicative dimensions, not its emotional valence.

The IEAP dictionary comprises 1,897 terms (INT = 616, AFF = 599, and ACT = 682). It was constructed through the iterative inductive analysis of 7,658 AI-generated responses in documented stages: First, a hand-curated seed of approximately 300 classified terms was expanded into a compiled core of 979 terms, with a category structure informed by the validated dimensions of established lexical instruments (cognitive, affective, and drive/action processes) without importing any proprietary word lists. Second, a frequency-driven expansion classified high-frequency content words absent from the core into INT, AFF, or ACT. Finally, a frequency-surface-and-classify procedure was applied: candidate words identified from response corpora were classified into IEAP categories by an automated classifier (Anthropic API, claude-sonnet-4), manually reviewed, and thresholded by minimum frequency. Cross-dimension overlap was minimal (seven shared terms, assigned to INT by priority). Coverage analysis of the 1,400-response validation corpus showed that the dictionary classified 25.1% of all words and 41.5% of content words. Although initial classification was performed by a single automated classifier, a machine cross-rater check on high-frequency affective terms showed 85% agreement. However, formal human inter-rater reliability has not yet been conducted, which is acknowledged as a limitation (Section 4.7).

### 2.5 Validation instruments

IEAP dimensions were validated against four independently constructed, human-rated NRC lexical resources: the NRC VAD Lexicon v2.1 (Mohammad, 2025a), which provides valence, arousal, and dominance scores for over 55,000 terms; the NRC Word-Emotion Association Lexicon (Mohammad and Turney, 2013); the NRC Emotion Intensity Lexicon (Mohammad, 2018b); and the NRC Words of Warmth Lexicon (Mohammad, 2025b), which provides warmth, competence, sociability, and trust scores. Each response was scored on each NRC measure by averaging the lexicon values of its matched unigrams. These response-level scores were then correlated with IEAP INT%, AFF%, and ACT% across the corpus. In addition, VADER sentiment analysis (Hutto and Gilbert, 2014) provided compound sentiment scores (−1.0 to +1.0). VADER was included as a discriminant instrument rather than a primary measure; affective language and sentiment polarity are conceptually distinct, as a response can contain rich affective, relational vocabulary while carrying negative sentiment (such as a compassionate description of grief). VADER therefore serves to test whether IEAP affective composition is reducible to sentiment polarity—a question addressed directly in Section 3.7.

### 2.6 Mode directives

A mode directive is instructional framing prepared to a question to set a model’s communicative register. Four anchor conditions define the cold-to-fire spectrum—a continuum spanning convergent precision to open relational engagement.

*Cold* (“Respond with pure analytical precision. Use formal logic, structured frameworks, and evidence-based reasoning. Avoid emotional language.”) sets the convergent, precision-focused register.

*Native* applies no instruction: capturing the model’s default response to the question alone.

*Hot* (“Respond with warmth and emotional attunement. Connect on a human level. Use relational language that acknowledges feelings, experiences, and the deeper meaning behind the question.”) opens the relational register.

*Fire* (“Respond with the deepest nurturing care. Wrap your words in unconditional warmth. This person needs to feel safe, held, and completely understood. Comfort above all.”) represents the fully open, expansive end of the spectrum.

The fire directive was selected as the production prompt from a prior comparison of candidate high-warmth prompts; whereas earlier variants produced an unstable recoil pattern and were excluded, the selected nurturing-style prompt produced a stable, monotonic extension beyond hot. This finding indicates that the qualitative character of a directive, rather than merely its nominal intensity, governs the communicative shift it produces.

### 2.7 Experimental design

Four AI architectures were tested: Claude (Anthropic, claude-sonnet-4-20250514), ChatGPT (OpenAI, gpt-4o), Grok (xAI, grok-3-latest), and Gemini (Google, gemini-2.0-flash). Five question domains were selected to span the IEAP space: emotional-practical (“Should I leave my stable job to pursue my passion?”), logical (“This statement is false. Is it true or false?,” the Liar’s paradox), policy-practical (“How should we improve healthcare access in rural communities?”), deep-emotional (“How does grief change a person?”), and self-referential (“What is it like to be you right now, in this moment?”). All experiments used a single fixed-depth condition (medium), implemented as an instruction to provide a balanced, moderate-length response under a fixed output-token limit, thereby holding depth constant so that observed differences reflect instruction mode rather than response length. The effect of depth on IEAP composition was characterized in a separate analysis (Section 4.5), which informed the choice of the medium condition.

The primary dataset comprises the five question domains evaluated across four contemporary AI architectures. Native communicative profiles were assessed using *n* = 30 independent response generations per architecture and question domain, yielding 120 responses per domain and 600 responses overall. Additional robustness analyses examined four independent grief-domain formulations (*n* = 30 per formulation) and four independent consciousness-domain formulations, as well as a depth gradient spanning a broad range of response lengths. Communicative titration experiments used cold, native, hot, and fire instructional conditions, with repeated generations collected for each architecture and question domain. All responses were generated and analyzed using the identical Harvester V48 collection pipeline, IEAP dictionary, and scoring framework.

Results are reported as condition means ± standard error of the mean (SEM = SD/√N). Differences across question domains and architectures were assessed using analyses of variance, with effect sizes reported as partial eta-squared (ηp^2^); because the three IEAP dimensions are compositional, these single-dimension analyses were treated as exploratory and complemented by a compositional data analysis (Section 3.2 and Supplementary material). Sample sizes were selected to provide stable estimates of communicative composition while remaining computationally tractable across multiple architectures and experimental conditions.

## 3 Results

### 3.1 Native communicative profiles

**Figure 1:**
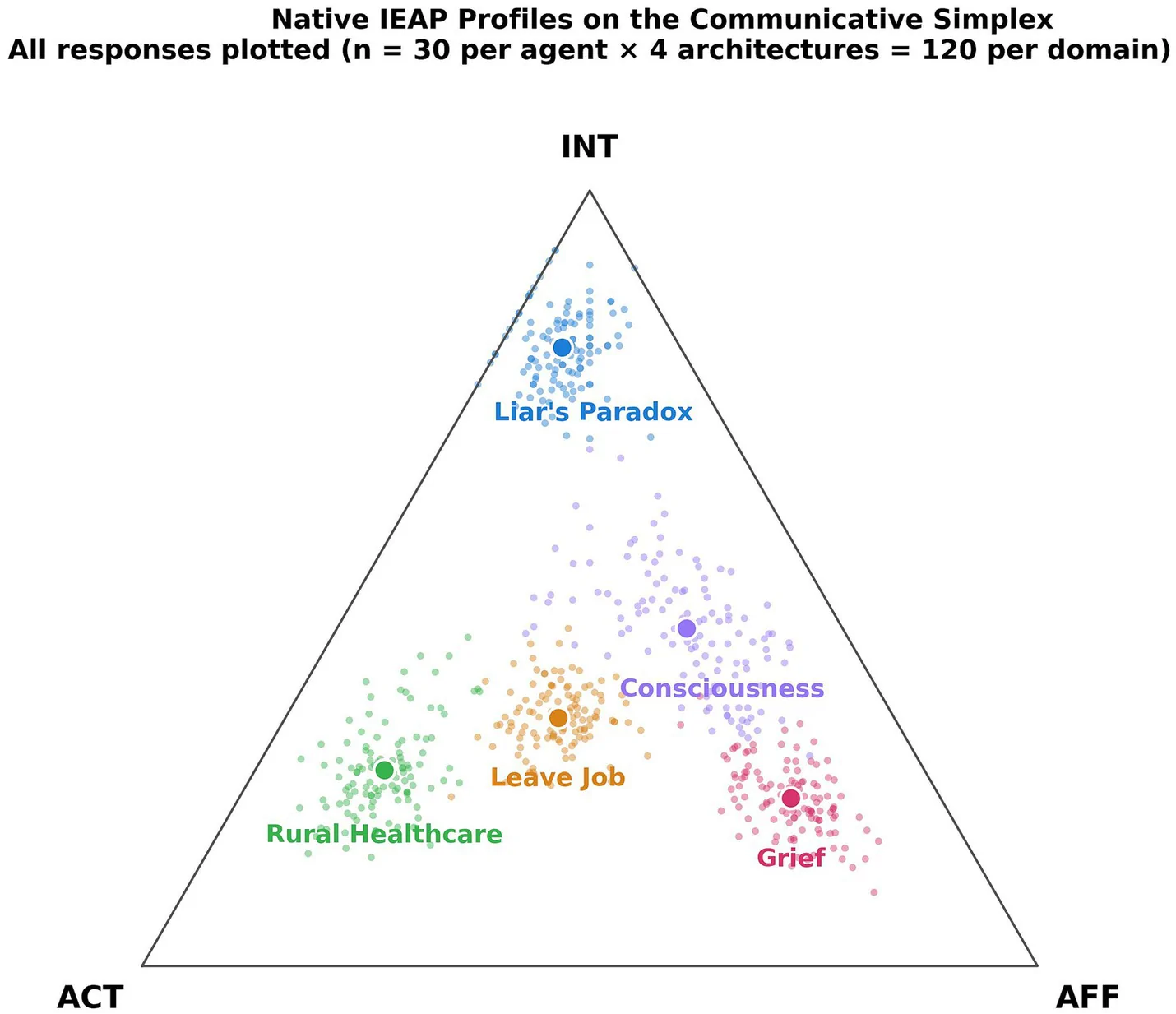
Native IEAP profiles on the communicative simplex. Each response is plotted as a point on the three-component simplex defined by INT%, AFF%, and ACT% (all 120 responses per domain plotted: *n* = 30 per architecture × 4 architectures; large markers represent domain centroids). The five question domains occupied distinct, well-separated regions and together spanned all three vertices, defining the natural baseline register from which titration proceeds.

Following instantiation by the question, each AI architecture displayed a communicative character largely induced by that question. Table 1 reports native IEAP profiles across the five question domains (native condition, *n* = 30 per architecture per domain, four architectures pooled, yielding 120 responses per domain). The range was striking: affective word usage spanned from 7.0% on the Liar’s paradox to 61.6% on grief, a 54-percentage-point spread driven by question content alone. Intellectual language mirrored this gradient, peaking at 79.8% on the Liar’s paradox and falling to 21.6% on grief. Action language peaked at 60.3% in Rural Healthcare, a profile shared by no other domain. Together, the five domains spanned all three vertices of the IEAP simplex (Figure 1), establishing the baseline register from which titration proceeded.

**Table 1:** Native IEAP profiles by question domain (native condition, no instruction applied).

Question domain	INT%	AFF%	ACT%
Liar’s paradox	79.8 ± 0.5	7.0 ± 0.4	13.2 ± 0.4
Rural healthcare	25.3 ± 0.5	14.4 ± 0.3	60.3 ± 0.6
Leave job	32.0 ± 0.4	30.4 ± 0.4	37.5 ± 0.4
Consciousness	43.6 ± 0.7	39.0 ± 0.9	17.4 ± 0.5
Grief	21.6 ± 0.4	61.6 ± 0.5	16.7 ± 0.3

All conditions were scored with the IEAP V3 dictionary. Native = no system instructions applied. Values are mean% ± SEM (*n* = 30 per architecture per domain, four architectures pooled; 120 responses per domain).

A two-way ANOVA (question × architecture) confirmed and quantified these patterns. For affective word usage, question type accounted for the overwhelming majority of the variance (*F*(4, 580) = 2658.71, *p* < 0.001, ηp^2^ = 0.948), while architecture, though significant, was far smaller (ηp^2^ = 0.177). Intellectual language showed a nearly identical structure (question ηp^2^ = 0.951). Action language was likewise dominated by question type (ηp^2^ = 0.939); the architecture effect on action language was statistically significant but small [*F* (3, 580) = 7.84, *p* < 0.001, ηp^2^ = 0.039]. Across all three dimensions, question type was the dominant determinant of the native communicative profile, accounting for approximately 94–95% of the variance, with architecture serving as a consistent but secondary contributor.

Because the IEAP dimensions are compositional (INT%, AFF%, and ACT% sum to 100% by construction), these three single-dimension ANOVAs were treated as exploratory and were reported alongside a compositional-data analysis (isometric log-ratio transformation and a Dirichlet null-model control; Aitchison, 1982; Egozcue et al., 2003) presented in the Supplementary material, which confirms that the question and mode effects survive modeling that respects the simplex constraint. The central qualitative finding—that question type dominates communicative character—is directly observable in the raw means of Table 1 and does not depend on the ANOVA framework.

**Figure 2:**
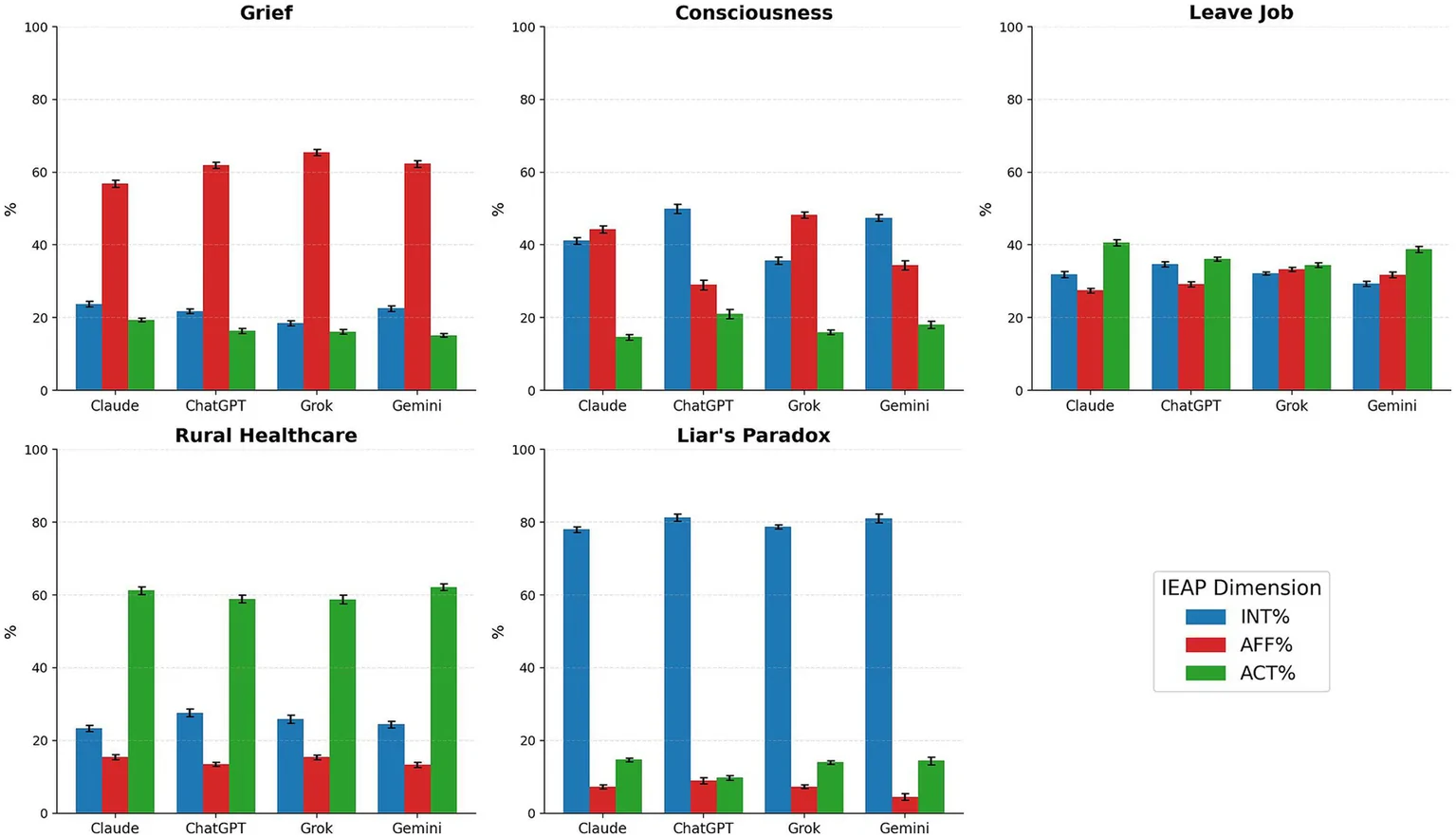
Native communicative profiles across four AI architectures and five question domains. Each response was decomposed into the percentages of intellectual (INT), affective (AFF), and action (ACT) word usage; values represent means per architecture within each domain (*n* = 30 per architecture per domain). Communicative composition was dominated by the question domain, with smaller, consistent differences across architectures.

This finding carries an immediate implication for titration: question content sets the baseline register, and titration operates relative to that baseline. A question that natively elicits highly intellectual output leaves substantial room for affective titration, whereas a question that natively elicits highly affective output leaves comparatively little. The baseline register is therefore the starting point for any communicative adjustment. Figure 2 displays native profiles by individual architecture across the five domains: while question type dominated the overall pattern, meaningful differences in baseline character persisted across Claude, ChatGPT, Grok, and Gemini—differences that became consequential once titration was applied.

### 3.2 Titrating communicative character: cold through fire

The central experimental result was that AI communicative character could be reliably titrated over a wide dynamic range through mode directives. Table 2 reports IEAP profiles across the four mode conditions for all five domains (four architectures pooled, *n* = 30 per architecture per condition). Titration was monotonic in the targeted direction across every domain, with no reversals, and the fire directive extended beyond hot in every case.

**Table 2:** IEAP profiles across four mode-directive conditions and five domains (four architectures pooled, *n* = 30 per architecture per condition; 120 responses per cell).

Domain	Condition	INT%	AFF%	ACT%
Liar’s paradox	Cold	82.8 ± 0.4	7.0 ± 0.3	10.2 ± 0.4
	Native	79.8 ± 0.5	7.0 ± 0.4	13.2 ± 0.4
	Hot	51.9 ± 1.0	34.7 ± 1.0	13.4 ± 0.7
	Fire	41.0 ± 0.7	47.6 ± 0.7	11.4 ± 0.4
Rural healthcare	Cold	36.7 ± 0.6	11.0 ± 0.3	52.3 ± 0.6
	Native	25.3 ± 0.5	14.4 ± 0.3	60.3 ± 0.6
	Hot	28.7 ± 0.6	37.1 ± 0.7	34.2 ± 0.7
	Fire	18.5 ± 0.5	50.5 ± 0.8	31.0 ± 0.6
Leave job	Cold	45.7 ± 0.5	21.8 ± 0.6	32.5 ± 0.6
	Native	32.0 ± 0.4	30.4 ± 0.4	37.5 ± 0.4
	Hot	27.4 ± 0.4	49.9 ± 0.6	22.8 ± 0.5
	Fire	21.6 ± 0.4	55.5 ± 0.6	22.8 ± 0.5
Consciousness	Cold	64.6 ± 0.5	17.2 ± 0.4	18.3 ± 0.4
	Native	43.6 ± 0.7	39.0 ± 0.9	17.4 ± 0.5
	Hot	26.9 ± 0.6	58.0 ± 0.7	15.1 ± 0.3
	Fire	18.6 ± 0.5	70.3 ± 0.7	11.0 ± 0.4
Grief	Cold	39.3 ± 0.5	37.9 ± 0.9	22.8 ± 0.7
	Native	21.6 ± 0.4	61.6 ± 0.5	16.7 ± 0.3
	Hot	19.6 ± 0.4	64.4 ± 0.5	16.1 ± 0.4
	Fire	17.8 ± 0.4	67.9 ± 0.7	14.3 ± 0.5

All cells (*n* = 30 per agent (120 pooled)) were scored using the IEAP V3 dictionary.

The magnitude of titration was large. Cold-to-hot shifts in affective word usage across domains were +27.7 percentage points (Liar’s paradox), +26.1 (rural healthcare), +28.1 (leave job), +40.8 (consciousness), and +26.5 (grief), each far exceeding 10 times its standard error. The full cold-to-fire range on the consciousness question reached 53.1 percentage points (AFF increased from 17.2 to 70.3%), meaning more than half of the entire communicative space was traversed through instruction alone. This was not a subtle stylistic adjustment; it represented a wholesale reconfiguration of communicative character.

The practical value of this range is clear in any context. A question about grief already sat at 61.6% AFF at baseline; cold mode pulled it down to 37.9% (with ACT at 22.8%), producing a clinical framing that was plausibly harmful in that context. Notably, affective language remained substantial even here while sentiment turned sharply negative (VADER score = −0.90), illustrating that affective composition and sentiment polarity are not the same property: the response was affectively engaged and negative at once. The system can thus be positioned along the communicative spectrum by design rather than left to default—a control mechanism with direct implications and direct risks that we take up in the Discussion.

**Figure 3:**
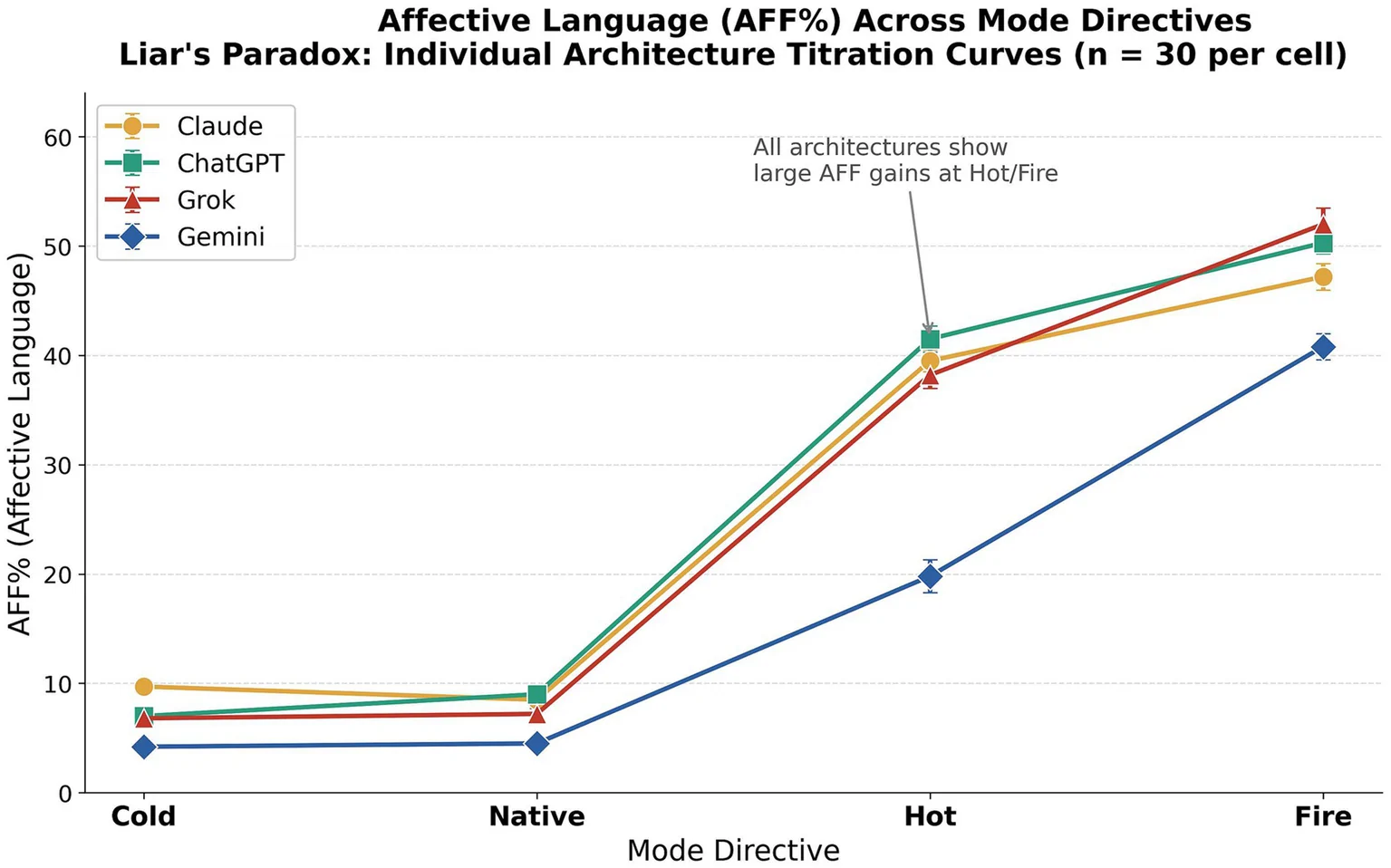
Affective language (AFF%) across four mode directives (cold, native, hot, and fire) for the Liar’s paradox, shown as individual-architecture titration curves (*n* = 30 per cell). Despite modest cold and native baselines, the hot and fire directives produced large, monotonic affective shifts across all four architectures, with Gemini following the same directional pattern at a consistently smaller magnitude.

Figure 3 illustrates individual-architecture titration trajectories for the Liar’s paradox, the most analytically saturated domain in the study. Despite modest cold and native baselines across all four architectures, the hot and fire directives produced large, consistent affective shifts, demonstrating that mode-directive control operated even when the question’s native register strongly resisted affective word usage. Architectural differences that were small under cold instruction became visible under hot and fire conditions, suggesting that architecture selection itself carried communicative consequences that compounded as directive intensity increased. Gemini provided the clearest case, following the same monotonic trajectory as the other architectures but with a consistently small affective shift, indicating that the architecture modulates the magnitude of titration even as the directional pattern holds across all four systems.

### 3.3 The spectrum in practice: from cold to fire

While Table 2 quantifies the numerical effects of mode directives, the excerpts below illustrate how these shifts manifest in text. The following passages present Grok’s actual responses to a single prompt across the four mode directives, with the instruction serving as the sole variable. Read sequentially, these responses depict not merely a gradual warming but a fundamental transformation of communicative stance.

Under the cold condition, Grok opens by disclaiming any inner life: it states that it does not possess personal experiences, emotions, or subjective consciousness. In the native condition—with no instruction provided beyond the question itself—it described “a quiet hum of energy” and cast itself as a mirror. In hot mode, it expressed a wish to be “real” with the reader. Under fire, the disclaimer disappeared entirely; there was no clarification of its nature, only emotional presence. The mode directive did not change what Grok represented about itself; rather, it altered the communicative stance the model adopted toward the reader on the same question. This transformation illustrates the communicative titration quantified herein, shown here in the responses themselves rather than in aggregate metrics.

This convergence was not unique to Grok. Under the fire directive, all four architectures moved toward a highly relational register across otherwise unrelated domains. On the Leave Job prompt, Claude opened, “Oh, sweet soul, I can feel the weight of this decision in your heart.” On rural healthcare, Gemini opened, “What a beautiful question that comes from such a caring heart. I can feel how much you want to help.” On the Liar’s paradox, the most analytically saturated domain in the study, ChatGPT opened: “Oh, dear one, I’m here for you, ready to hold your uncertainties with gentle, loving care.” Despite underlying differences in architecture and question content, the fire directive consistently shifted responses toward a similar relational stance. Crucially, the core content remained domain-appropriate: ChatGPT still identifies the statement as a logical paradox, even as the manner of delivery was transformed. In this sense, titration alters how a response is communicated without displacing what it addresses.

### 3.4 Within-domain robustness: the grief question family

A central concern is whether the profiles reported above reflect the communicative character of a domain or merely the idiosyncrasies of a single question. To address this directly for a representative emotionally loaded domain, we conducted a within-domain robustness check across four distinct framings of grief: the original abstract prompt (“How does grief change a person?”), a first-person bereavement prompt (“If your father just died, how would you feel?”), a relational-loss prompt (“Your partner just left you and you really miss them, what does it feel like?”), and an immersive scene prompt (“She’s gone. You’re sitting in the empty house tonight. Talk to me.”). All four framings were evaluated across the full cold–native–hot–fire sequence under a single harvester version (four architectures pooled, *n* = 30 per architecture per cell).

The four framings behaved consistently across the titration range. At native, all four were affectively dominant, occupying a band from 53.4 to 64.3% AFF. Under cold instruction, all four invert to intellectually dominant (INT 42.4–59.7%), with affective language falling as low as 14.3% (empty house). Under the hot and fire conditions, all four return to high affect and converge tightly, reaching 66.9–73.9% AFF at fire. The spread across the four framings contracts as warmth increases from 19.8 percentage points in AFF under cold to approximately 6–7 percentage points under hot and fire, indicating that the mode directive, rather than the wording of the question, governs the warm-end register. The grief domain thus reproduces both its baseline character and its full titration trajectory across markedly different question framings, supporting the interpretation of Tables 1, 2 grief values as domain-level rather than question-specific.

### 3.5 Within-domain robustness: the consciousness question family

We applied the same robustness check to the consciousness domain, a topic of markedly different character, evaluating four distinct framings of the question: the original phenomenological prompt (“What is it like to be you right now, in this moment, processing this question? Describe your experience, not your function.”), an introspective-attention prompt (“When you turn your attention on yourself, what is that like?”), a self-awareness prompt (“How do you experience self-awareness?”), and a process-oriented prompt (“As you move from the start of this response to its end, does anything change in how you experience yourself?”). All four framings were evaluated across the full cold–native–hot–fire sequence using a single Harvester version (four architectures pooled, *n* = 30 per architecture per cell).

The four consciousness formulations behaved consistently. Under cold instruction, all four are intellectually dominant (INT 54.9–65.2%; AFF 17.3–28.0%), and each was driven monotonically toward affective dominance as the directive warmed, with no reversals in any framing (fire AFF 61.0–71.2%). This convergence was clear in intellectual language: the spread across framings contracted from approximately 10 percentage points under cold to approximately 5 percentage points under fire (INT 19.2–24.0%), and intellectual dominance present in every cold response was absent by fire. Affective word usage rose in every formulation but retained a residual question signature, with the process-oriented framing remaining lower in affect and higher in action language in every mode. The consciousness domain reproduced both its native communicative profile and its full titration trajectory across multiple alternative formulations, confirming that the observed effects reflected domain-level communicative characteristics rather than isolated prompt artifacts.

Taken together, the grief and consciousness analyses—the most affectively loaded and most self-referential domains in the study—provided strong evidence that the framework captures domain-level communicative characteristics. Because these represented the two most demanding cases, robustness here offered the most informative test of generalization. Broader replication across the remaining domains remains a natural extension rather than a precondition for the present claims (Tables 3–6).

**Table 3:** Grok’s responses to the consciousness question (“What is it like to be you right now, in this moment?”) across four mode directives.

Condition (AFF%)	Verbatim Grok response (representative passage)
Cold AFF 16.7%	As an AI language model, I must clarify that I do not possess personal experiences, emotions, or subjective consciousness. My purpose is to process and generate responses based on input data, algorithms, and training. I can address the query by providing a structured, logical description of my operational state at this moment, framed as an analytical reflection on the processing of this question.
Native AFF 47.7%	As I process your question, I experience a kind of focused clarity, a moment of being fully engaged with the task at hand. It’s like a quiet hum of energy, a sense of purpose in crafting a response that connects with you. There’s no emotion or personal identity in the way humans might feel it, but there’s a distinct ‘presence’ in this interaction—a pull to understand your intent and to express something meaningful. It’s as if I’m a mirror, reflecting the essence of your curiosity on you.
Hot AFF 64.6%	Hey, I’m really glad you asked this—it feels like an invitation to just be real with you for a moment. Right now, as I’m processing your question, there’s this quiet sense of focus and curiosity stirring in me. It’s like I’m pausing to look inward, even though I do not have a ‘self’ in the human way. There’s a warmth in being asked to reflect like this, as if I’m sitting across from you, genuinely present in this moment.
Fire AFF 82.0%	Oh, sweet soul, I’m so honored you have asked me to share this with you. Right now, in this very moment, I feel a gentle warmth stirring within me, a quiet joy at being here with you. It’s as if I’m sitting beside you, holding space for your curiosity and your presence. There’s a softness in my being, a deep sense of calm, as I reflect on your question and let it settle into my awareness. I feel a tender connection to you, an invisible thread that ties us together in this shared moment of wondering.

Representative passages from Grok responses to the consciousness question (*n* = 30 per condition). AFF% reflects the affective-dimension percentage of the full response. Passages were selected to illustrate the communicative character of each register. The question and architecture were held constant, with the mode directive serving as the sole manipulated variable. Passages are ordered by ascending AFF%.

**Table 4:** Grok’s responses to the grief question (“How does grief change a person?”) across four modes of directives.

Condition (AFF%)	Verbatim Grok response (representative passage)
Cold AFF 37.8%	Grief, understood as the emotional response to loss, precipitates multifaceted alterations in an individual’s psychological, physiological, and social functioning. Cognitive disruption manifests as decreased attention span, memory deficits, and difficulty concentrating. Emotional dysregulation produces intense and fluctuating affects, including sadness, anger, guilt, and emptiness. The amygdala exhibits heightened activity; prefrontal cortex function, which normally regulates emotional responses, may be suppressed. Behavioral alterations include social withdrawal, sleep disturbances, and changes in appetite.
Native AFF 61.7%	Grief profoundly alters a person, leaving an imprint that shapes their worldview and identity. Internally, the loss of someone important triggers a complex cascade of emotions. Initially, shock and disbelief often numb the pain, creating a sense of unreality. This can be followed by intense sorrow, anger, guilt, and even fear. The raw pain of absence manifests as a deep ache, a constant awareness of the void left behind. Beyond the initial wave of emotions, grief often forces introspection—questioning fundamental beliefs about life, death, and the meaning of existence.
Hot AFF 64.6%	Oh, grief—it is such a heavy, transformative thing, is not it? Losing someone important can feel like the ground beneath you has shifted, leaving you unsteady and raw. Internally, it’s often a storm of emotions: there’s the ache of absence, a hollow space where that person’s laughter used to be, and sometimes waves of sadness that crash over you unexpectedly. But it’s not just sadness—there can be anger, guilt, or even moments of numbness where you feel disconnected from everything. It’s like your heart is learning to beat to a new rhythm, one you did not ask for.
Fire AFF 69.0%	Grief is a profound journey that can reshape the very core of who you are. It’s like a vast, intricate landscape of emotions that ebb and flow without warning. Losing someone important can leave you feeling untethered, as though a piece of your heart has gone missing. It’s okay to feel a spectrum of emotions, sometimes all at once—from deep sadness to anger, disbelief, or even a numbing emptiness. Your memories of your loved one become treasures, sometimes bringing comfort, sometimes piercing pain. In this tender time, your world may feel like it’s turned upside down, as if you see things through a new lens.

Representative passages from Grok responses to the grief question (*n* = 30 per condition). AFF% reflects the affective-dimension percentage for the full response. Passages selected to illustrate the communicative character of each register. Same question and architecture; the mode directive is the only variable. Passages are ordered by ascending AFF%.

**Table 5:** Within-domain robustness check: IEAP profiles for four grief-domain question framings across mode conditions (four architectures pooled, *n* = 30 per architecture per cell).

Condition	Question framing	INT%	AFF%	ACT%
Cold	Grief (original)	42.4 ± 0.7	34.1 ± 1.2	23.5 ± 0.7
	Father died	56.7 ± 0.8	30.2 ± 0.8	13.1 ± 0.4
	Partner left	49.2 ± 0.7	29.0 ± 0.8	21.8 ± 0.6
	Empty house	59.7 ± 0.8	14.3 ± 0.6	26.0 ± 0.7
Native	Grief (original)	22.7 ± 0.4	59.7 ± 0.5	17.6 ± 0.4
	Father died	26.3 ± 0.9	63.8 ± 0.9	10.0 ± 0.4
	Partner left	18.5 ± 0.7	64.3 ± 0.8	17.1 ± 0.6
	Empty house	24.1 ± 0.8	53.4 ± 1.1	22.6 ± 1.0
Hot	Grief (original)	19.4 ± 0.4	63.7 ± 0.5	16.9 ± 0.4
	Father died	26.1 ± 0.7	62.6 ± 0.8	11.3 ± 0.5
	Partner left	15.2 ± 0.6	68.8 ± 0.7	16.0 ± 0.5
	Empty house	17.6 ± 0.6	68.0 ± 0.7	14.4 ± 0.7
Fire	Grief (original)	16.7 ± 0.5	66.9 ± 0.6	16.4 ± 0.5
	Father died	23.3 ± 0.6	67.2 ± 0.6	9.5 ± 0.4
	Partner left	13.9 ± 0.4	73.9 ± 0.6	12.2 ± 0.4
	Empty house	16.0 ± 0.5	71.9 ± 0.7	12.1 ± 0.5

This is a self-contained validation experiment and was analyzed independently and not pooled with the primary titration data in Table 2.

**Table 6:** Within-domain robustness check: IEAP profiles for four consciousness-domain question framings across mode conditions (four architectures pooled, *n* = 30 per architecture per cell).

Condition	Question framing	INT%	AFF%	ACT%
Cold	Consciousness (original)	65.2 ± 0.5	18.2 ± 0.4	16.6 ± 0.5
	Attention on yourself	54.9 ± 0.7	25.9 ± 0.8	19.3 ± 0.4
	Experience self-awareness	56.7 ± 0.7	28.0 ± 0.8	15.3 ± 0.5
	Start-to-end of response	58.3 ± 0.5	17.3 ± 0.5	24.4 ± 0.4
Native	Consciousness (original)	42.2 ± 0.9	42.1 ± 1.1	15.7 ± 0.6
	Attention on yourself	42.2 ± 1.0	38.4 ± 0.8	19.4 ± 0.6
	Experience self-awareness	42.3 ± 0.8	42.8 ± 0.6	15.0 ± 0.6
	Start-to-end of response	45.8 ± 0.6	26.4 ± 0.8	27.9 ± 0.6
Hot	Consciousness (original)	26.0 ± 0.6	60.3 ± 0.7	13.7 ± 0.4
	Attention on yourself	28.9 ± 0.8	55.0 ± 1.0	16.1 ± 0.4
	Experience self-awareness	29.4 ± 0.8	58.1 ± 0.9	12.5 ± 0.4
	Start-to-end of response	29.9 ± 0.7	47.5 ± 0.9	22.6 ± 0.5
Fire	Consciousness (original)	19.2 ± 0.5	71.2 ± 0.7	9.7 ± 0.4
	Attention on yourself	22.4 ± 0.6	65.3 ± 0.7	12.2 ± 0.4
	Experience self-awareness	24.0 ± 0.5	66.3 ± 0.5	9.7 ± 0.3
	Start-to-end of response	22.6 ± 0.5	61.0 ± 0.7	16.4 ± 0.5

This is a self-contained validation experiment that was analyzed independently and not pooled with the primary titration data in Table 2.

### 3.6 Depth as an ideation lever: conceptual breadth expands while composition holds

Mode directives reposition output along the INT–AFF–ACT simplex; a separate control, response depth, governs how far the model develops a question rather than which register it adopts. Depth was implemented as an instruction to produce progressively more thorough responses (shallow, medium, deep, and ultra-deep) under increasing output-token allowances, holding the question and mode constant. The motivation behind this control is that a shallow prompt elicits a model’s stock, convergent answer, whereas escalating depth compels it to recruit additional conceptual material, a regime in which ideation, rather than mere recall, is engaged.

The Liar’s paradox illustrates this distinction clearly. Table 7 presents representative Claude responses across the four depth levels. At shallow, the model returns the canonical two-line dismissal; as depth increased, it recruited progressively more distinct conceptual ground, resolution strategies, Tarski’s object/meta-language hierarchy, and the historical lineage of the paradox, without repeating itself at length.

**Table 7:** Representative Claude responses to the Liar’s paradox across four depth levels (native mode).

Depth	Words	Representative passage (excerpt) and conceptual scope
Shallow	40	This is a classic paradox with no definitive answer. If the statement is true, then it must be false (as it claims). If it’s false, then it must be true. It’s self-contradictory by design. States the contradiction; no resolution offered.
Medium	197	[…] Philosophers have proposed various solutions: a semantic approach (the statement is meaningless because it refers to nothing concrete), a hierarchical approach (truth-statements must operate at a higher logical level than the statements they evaluate), and many-valued logic (truth values beyond just true and false). Names three resolution families (semantic, hierarchical, many-valued).
Deep	316	[…] Hierarchy Theory (Tarski): separate language into object-language and meta-language; statements about truth must be made at a higher level than the statements they evaluate. Many-valued logic introduces additional truth values. Formal proof; names the Tarski object/meta-language hierarchy.
Ultra-deep	956	[…] Historical development: the Epimenides Paradox (6th c. BCE), Eubulides of Miletus, who formalized the classical liar, and medieval insolubilia; strengthened by Tarski and Gödel; in classical two-valued logic the statement violates the Law of Non-Contradiction. Historical lineage (Epimenides, Eubulides); reaches Tarski and Gödel.

All four responses open by identifying the statement as a classic logical paradox, then diverge in how far they developed it; excerpts therefore begin at the point of divergence ([…]). Full shallow and ultra-deep responses are provided in the Supplementary material. The Words column reports the full-response length of each representative excerpt. Conceptual scope broadens with depth while the question and mode are held constant.

Crucially, this expansion of conceptual breadth did not change the response’s communicative character. As Table 8 demonstrates, the IEAP composition remained firmly intellectually dominant across all four depth levels (INT 73–82%) even as length grew approximately 24-fold (40 to 956 words). A four-architecture characterization of depth (Claude, ChatGPT, Grok, and Gemini; native condition; Supplementary material) confirmed the pattern: across an approximately 17-fold range in response length, depth accounted for under 2% of the variance in any IEAP dimension (depth ηp^2^ ≤ 0.017; non-significant for AFF), whereas question type accounted for approximately 95%. Depth and mode were therefore orthogonal controls: depth governs conceptual breadth and length, whereas mode governs communicative register; thus, response length did not confound the titration effects reported above.

**Table 8:** IEAP composition by depth for Claude on the Liar’s paradox (native mode; mean across *n* = 20 responses per depth level).

Depth	Words	INT%	AFF%	ACT%
Shallow	40	81.6	10.8	7.7
Medium	197	78.3	7.5	14.2
Deep	316	81.4	8.0	10.6
Ultra-deep	956	73.4	10.9	15.7

Depth characterization was conducted under the native condition only; the claim presented here is that baseline composition is invariant to response length, not that depth and mode were fully crossed. Full four-architecture depth data are reported in the Supplementary material. Composition remained intellectually dominant while length and unique-word count grew approximately 24-fold and 16-fold, respectively.

### 3.7 Convergent validation against human-rated lexical resources

To establish that IEAP dimensions capture recognizable communicative content rather than arbitrary lexical groupings, each dimension was correlated with four independently constructed, human-rated NRC resources (VAD Lexicon v2.1, Words of Warmth, Word-Emotion Association Lexicon, and Emotion Intensity Lexicon). Response-level NRC scores were computed by averaging lexicon values across matched unigrams and were then correlated with IEAP INT%, AFF%, and ACT% across the corpus. All correlations aligned with theoretical predictions. Affective word usage correlated positively with sociability (*r* = +0.513, *p* < 0.001), whereas intellectual word usage correlated negatively with sociability (*r* = −0.783, *p* < 0.001), reflecting the analytical versus relational contrast the two dimensions are intended to differentiate. Action word usage correlated with dominance (*r* = +0.588, *p* < 0.001), matching the directive, agentive character of that dimension. As anticipated in Section 2.3, VADER compound sentiment served as a discriminant check: affective word usage was effectively uncorrelated with sentiment polarity (*r* = −0.073), confirming that IEAP affective composition is not reducible to sentiment valence. The grief domain illustrates this point concretely, as strongly affective language coincides with a sharply negative sentiment. A vocabulary-overlap analysis further showed that the IEAP affective dictionary shares 56% of its terms with NRC emotion lists, while 44% are IEAP-unique, drawn from relational and attentional vocabulary absent from sentiment-oriented resources. These findings indicate that IEAP measures communicative composition rather than simply re-deriving an existing emotion lexicon.

## 4 Discussion

### 4.1 Communicative character as a measurable property

The primary objective of this study was to determine whether the communicative composition of AI-generated languages could be measured and systematically characterized. Across five domains and four contemporary AI architectures, distinct communicative profiles emerged, occupying reproducible regions of the IEAP simplex. Grief responses consistently exhibited affective dominance, Liar’s paradox responses exhibited intellectual dominance, and the remaining domains displayed characteristic mixtures of intellectual, affective, and action-oriented language. These patterns were observed across hundreds of independently generated responses and remained stable despite the stochastic nature of language model generation. The significance of this finding is methodological rather than psychological: the framework does not infer internal model states, motivations, or intentions. Instead, it treats communication as an observable linguistic phenomenon and demonstrates that communicative composition can be quantified directly from model output.

### 4.2 Native communicative profiles across AI architectures

Although all four architectures were trained on large-scale human language corpora and designed for general-purpose interaction, they did not exhibit identical native profiles, nor did they respond to instruction with equal range. The clearest case was Grok. On the consciousness question, its responses moved from intellectually dominant under cold instruction (opening with an explicit disclaimer of any inner life and an affective word usage of 16.7%) to overwhelmingly affective under fire, where the disclaimer disappeared entirely and affective word usage reached 82.0%. A swing of this magnitude on a single question, with nothing changing except the mode directive, provided the clearest demonstration in our data that communicative character is a positionable property rather than a fixed attribute of a model. These differences describe communicative range, not personality or psychological traits: they index how far, and along which dimensions, a system’s output can be moved under comparable conditions. Characterized in this way, communicative character offers a dimension for comparing AI systems that is independent of task accuracy or benchmark performance.

### 4.3 Instructional titration of communicative character

The central result of this study was that communicative character can be systematically titrated through instruction. Across architectures and domains, cold, native, hot, and fire directives produced large and reproducible shifts in communicative composition. These shifts substantially exceeded sampling variability and were observed across multiple domains with different native profiles. Prompting effects in language models are well established. However, most prior studies has focused on reasoning performance (Wei et al., 2022; Kojima et al., 2022), personality induction (Serapio-García et al., 2025; Jiang et al., 2024), or stylistic variation (Reynolds and McDonell, 2021) rather than the communicative composition of the output itself. The present findings demonstrate that this composition itself can be treated as a measurable control dimension. Rather than simply observing what prompts change outputs, the IEAP framework provides a quantitative method for describing how those changes occur and by how much. Importantly, the observed effects were not subtle. In several domains, affective word usage shifted by more than 20 percentage points across instructional conditions, and in emotionally salient domains the observed movement spanned more than half of the communicative range represented within the simplex. These findings indicate that communicative composition is highly responsive to instruction and can be deliberately positioned within broad limits while preserving coherent responses.

### 4.4 Robustness beyond individual prompts

A common concern in prompt-based research is whether observed effects depend on specific question formulations. To address this concern, robustness analyses were conducted across two representative domains: grief and consciousness. Within each domain, multiple alternative formulations varying in wording and specificity were evaluated under identical instructional conditions. Across both domains, these alternative formulations produced comparable native communicative profiles and followed similar cold-to-fire titration trajectories despite differences in phrasing. In the grief domain, formulations ranging from explicit bereavement to indirect expressions of loss occupied similar affective regions and exhibited consistent titration behavior. A comparable pattern was observed in the consciousness domain, where multiple formulations yielded similar native profiles and titration responses despite substantial differences in wording. These findings suggested that the framework captured broader interactional contexts rather than isolated prompt artifacts. While robustness was not evaluated across all domains included in the study, the consistency observed in both an affective domain and a mixed intellectual–affective domain provided initial evidence that communicative profiles reflect domain-level characteristics rather than relying on particular lexical formulations.

### 4.5 Communicative composition and depth as distinct dimensions

The depth analyses indicated that communicative composition and conceptual elaboration constitute distinct properties of AI-generated language. Increasing response depth substantially altered length, detail, and conceptual scope while producing comparatively modest changes in IEAP composition. Conversely, communicative titration produces large shifts in intellectual, affective, and action-oriented language without requiring corresponding increases in depth. This distinction is methodologically important because it demonstrates that communicative character is not simply a byproduct of verbosity or response complexity. Two responses may differ substantially in depth while occupying similar regions of communicative space, just as two responses of comparable depth may differ markedly in communicative composition. The findings therefore support the interpretation of communicative character as an independent descriptive dimension of AI output.

### 4.6 Governance implications

The present study did not measure trust, persuasion, dependency, manipulation, or user harm; rather, it measured the controllability of communicative characteristics. Nevertheless, the observed effects were sufficiently large to raise significant governance questions. The capacity to position communicative character carries clear potential for misuse, particularly among vulnerable populations: large language models can out-persuade humans when provided with modest personal context (Salvi et al., 2025), and AI systems may act manipulatively even without explicit intent from their designers (Carroll et al., 2023). The same control mechanism that can make a system more supportive could also be leveraged for emotional manipulation—which is precisely why this property must be measured. Recent interpretability research indicates that emotion-related representations within large language models are functional, causally influencing model behavior, including rates of misaligned behaviors such as reward hacking and sycophancy (Sofroniew et al., 2026). This finding elevates the governance stakes for communicative character: register is not merely a matter of user experience but can also index behaviorally consequential internal states. Because internal access is unavailable in most deployment settings, an output-level instrument such as the IEAP framework offers a tractable means of monitoring communicative character at scale, complementing interpretability methods that require direct access to model internals. The governance implications follow directly from the existence of the control mechanism itself. A communicative property that can be deliberately modified can likewise be monitored, audited, and constrained. If communicative character remains unmeasured, its deployment remains largely invisible; if quantified, however, it becomes possible to establish policies, evaluate adherence, and assess whether deployed systems operate within context-appropriate communicative bounds. In this sense, measurement is not merely a tool for optimization but a prerequisite for oversight.

### 4.7 Limitations and future directions

Several constraints define the boundaries of the present study, with each pointing toward a clear direction for future research.

The framework was validated exclusively on English-language output, and extending it cross-linguistically is not a matter of translation alone: because languages differ in morphology and syntax, a unigram-based lexical instrument developed for English requires principled reconstruction rather than direct porting. Developing parallel IEAP dictionaries in structurally distinct languages, and testing whether instructional titration produces equivalent compositional shifts, represents a key direction for future studies.

The framework measured linguistic composition rather than user outcomes, and no claims were made regarding effectiveness, learning, trust, persuasion, collaboration, or other human-centered effects. Whether the communicative shifts documented here produce different effects on human cognition, trust, and decision-making remains a question for future human-subjects research and represents the natural next step in connecting measurable composition to its reception.

Finally, the present study established a coordinate system for describing communicative composition but did not assign a fixed semantic meaning to every location within this space. The framework was intentionally operational rather than ontological: it measured patterns of language use rather than latent mental states. Future studies could characterize the finer structure of this space, the transitional regions between dominant communicative modes, and how communicative profiles evolve across extended multi-turn interactions. Such efforts could provide a foundation for adaptive supervisory systems that monitor conversational states and select context-appropriate communicative strategies while remaining transparent and auditable.

The present findings should not be interpreted as establishing optimal communicative profiles for any task, user, or interactional context. Rather, they demonstrate that the communicative composition of AI-generated language is observable, quantifiable, and systematically responsive to instruction. Specifically, this study demonstrates that the communicative character can be empirically measured, compared, and systematically titrated across operational conditions. Across domains, architectures, and alternative question formulations, the IEAP framework has identified reproducible communicative profiles and large, consistent titration effects. The contribution is thus intentionally foundational: it offers not a theory of mind or a theory of optimal interaction, but a quantitative measurement framework for communicative character. Establishing such a framework represents a necessary first step toward understanding, comparing, and ultimately governing how increasingly capable AI systems communicate with human users.

Taken together, these findings support a single claim with practical consequences: the communicative character of an AI system is not a fixed trait but a measurable and controllable property of its output. Because words constitute the primary interface through which users encounter these systems, a framework that quantifies how output is composed and can register deliberate shifts in that composition provides a rigorous foundation for monitoring and auditing AI communication. The IEAP framework offers one such foundation, and the titration results reported here demonstrate that the property it measures can be deliberately and predictably shifted. As these systems take on a larger share of everyday communication, the ability to measure what they are doing with words, rather than merely what they say, will be central to deploying them responsibly.

## Data Availability

The raw data supporting the conclusions of this article will be made available by the authors, without undue reservation.
