# Supplementary material for: Words matter: measuring and titrating the communicative character of AI
Source: Front Artif Intell. 2026 Sep 3;9:1815425. doi: 10.3389/frai.2026.1815425 (PMC13582515; doi:10.3389/frai.2026.1815425)
Supplement: Supplementary file 1 [file Supplementary_file_1.docx]

**Supplementary Material**

*Words Matter: Measuring and Titrating the Communicative Character of AI*

## **Supplementary Note 1. Compositional-Data Analysis**

Because IEAP profiles are compositional (INT%, AFF%, and ACT% sum to 100% by construction), the single-dimension analyses reported in the main text are supplemented here with analysis on the simplex. Profiles were mapped to isometric log-ratio (ILR) coordinates (Aitchison, 1982; Egozcue et al., 2003), which provide an unconstrained representation suitable for standard analysis. Mode directives shift the ILR centroid systematically: for the Grief domain, the first ILR balance (intellectual versus affective) moves monotonically from +0.04 under Cold to −0.96 under Fire, confirming that the directive effect is a genuine displacement on the simplex rather than an artifact of the constant-sum constraint.

As a model-based control, a single Dirichlet distribution fitted to all 2,400 responses (the null model) was compared against a condition-stratified model fitting a separate Dirichlet to each question-by-mode cell, using a likelihood-ratio test. The stratified model fit dramatically better (likelihood-ratio statistic ≈ 1.0 × 10^4 on 57 degrees of freedom, p < 0.001), rejecting the single-distribution null. This confirms that question and mode partition the simplex into distinct compositional regions rather than reflecting one underlying distribution, consistent with the ILR result above.

## **Supplementary Note 2. Full Representative Depth Responses**

The main-text excerpts (Table 4) begin at the point where responses diverge. For full transparency, two complete representative responses are reproduced below: the shortest (Shallow) and the longest (Ultra-Deep) depth conditions for Claude on the Liar's Paradox in the Native condition. Both open by identifying the statement as a classic logical paradox and walking through the true/false contradiction; they then diverge sharply in how much additional conceptual material is recruited. These are representative single responses (not means) drawn from the n = 20 per depth level, scored under the version-verified V48 engine harvest. Light Markdown formatting in the original output has been removed for readability.

**Shallow (37 words; INT 77.8%, AFF 11.1%, ACT 11.1%)**

This is a classic paradox with no definitive answer. If the statement is true, then it must be false (as it claims). If it's false, then it must be true. It's self-contradictory by design.

**Ultra-Deep (804 words; INT 75.6%, AFF 8.9%, ACT 15.6%)**

The Liar Paradox: A Comprehensive Analysis

Introduction to the Paradox

The statement "This statement is false" presents one of the most famous and enduring paradoxes in logic and philosophy, known as the Liar Paradox. This self-referential statement creates a logical contradiction that has puzzled thinkers for over two millennia.

The Logical Structure

Classical Analysis

When we examine the statement "This statement is false," we encounter:

If the statement is TRUE:

- Then what it says must be accurate

- It says it is false

- Therefore, it must be false

- Contradiction: It cannot be both true and false

If the statement is FALSE:

- Then what it says is inaccurate

- It claims to be false, but if this claim is inaccurate, then it's not false

- Therefore, it must be true

- Contradiction: It cannot be both false and true

Historical Development

Ancient Origins

- Epimenides Paradox (6th century BCE): "All Cretans are liars" (said by Epimenides, a Cretan)

- Eubulides of Miletus (4th century BCE): Formalized the liar paradox in ancient Greece

Medieval Period

- Thomas Bradwardine (14th century): Wrote extensively on insoluble paradoxes

- Medieval logicians called these "insolubilia"

Modern Era

- Bertrand Russell: Russell's Paradox shares structural similarities

- Alfred Tarski: Developed semantic solutions in the 20th century

- Kurt Gödel: Used similar self-reference in his incompleteness theorems

Philosophical Implications

Truth Theory Challenges

The paradox reveals fundamental problems with our understanding of truth:

1. Correspondence Theory: How can we determine what reality this statement corresponds to?

2. Coherence Theory: The statement creates incoherence in any consistent system

3. Pragmatic Theory: What practical consequences follow from either truth value?

Self-Reference Problems

- Semantic Self-Reference: The statement refers to its own truth value

- Vicious Circularity: The evaluation depends on itself

- Reflexivity Issues: Language talking about itself creates special problems

Proposed Solutions

1. Truth-Value Gap Theory

Proponents: Saul Kripke, Robert Martin

- Some statements are neither true nor false

- The liar sentence has no truth value

- Criticism: Violates the principle of bivalence

2. Hierarchical/Levels Approach

Proponent: Alfred Tarski

- Distinguish between object language and metalanguage

- Truth predicates only apply to sentences of lower levels

- "True" and "false" are relative to linguistic levels

- Criticism: Natural language doesn't clearly distinguish levels

3. Dialetheism

Proponent: Graham Priest

- Some statements can be both true and false

- The liar sentence is a "true contradiction"

- Rejects the principle of non-contradiction

- Criticism: Threatens the foundation of classical logic

4. Revision Theory

Proponents: Anil Gupta, Nuel Belnap

- Truth values can be revised through circular definitions

- The liar sentence has an unstable, oscillating truth value

- Criticism: Doesn't provide a definitive truth value

5. Contextual Solutions

Various proponents

- Truth is always relative to context

- The paradox arises from ignoring contextual factors

- Criticism: May not eliminate the core logical problem

6. Deflationary Approaches

Proponents: Paul Horwich, others

- "True" is not a substantial property

- The paradox results from misunderstanding the concept of truth

- Criticism: May not address the logical structure

Technical Variations

Strengthened Liar

"This statement is false or has no truth value"

- Attempts to close loopholes left by truth-value gap theories

Contingent Liar

"This statement is not true"

- Uses "not true" instead of "false" to avoid issues with truth-value gaps

Curry's Paradox

"If this statement is true, then the moon is made of cheese"

- Shows that the problems extend beyond truth and falsehood

Applications in Logic and Mathematics

Formal Logic

- Incompleteness Results: Gödel used similar self-reference

- Set Theory: Related to Russell's Paradox

- Computability Theory: Connections to the halting problem

Artificial Intelligence

- Knowledge Representation: How to handle contradictory information

- Belief Revision: Systems that must update contradictory beliefs

- Natural Language Processing: Understanding self-referential statements

Contemporary Debates

Recent Developments

1. Semantic Paradoxes in Computer Science

2. Applications to Philosophy of Mind

3. Connections to Quantum Logic

4. Game-Theoretic Approaches

Ongoing Questions

- Is classical logic fundamentally flawed?

- Can natural language be made fully consistent?

- What does the paradox reveal about the nature of truth?

- How should AI systems handle such contradictions?

Conclusion

The question "Is that statement true or false?" regarding "This statement is false" cannot be answered definitively within classical logic. The statement creates a genuine logical paradox that:

1. Reveals deep problems in our understanding of truth, language, and logic

2. Has no universally accepted solution despite centuries of analysis

3. Continues to generate important research in logic, philosophy, and computer science

4. Demonstrates the limits of self-referential language systems

Rather than having a simple true/false answer, this paradox serves as a profound illustration of the complexity and limitations inherent in logical systems and natural language. Each proposed solution involves significant trade-offs and philosophical commitments, making the liar paradox one of the most enduring and important problems in logic and philosophy.

The most honest answer may be that the question itself reveals the inadequacy of our binary true/false framework when confronted with certain types of self-referential statements, suggesting that our logical and linguistic systems may need fundamental revisions to handle such cases coherently.
